# Specific role of *N*-methyl-D-aspartate (NMDA) receptor in elastin-derived VGVAPG peptide-dependent calcium homeostasis in mouse cortical astrocytes *in vitro*

**DOI:** 10.1038/s41598-019-56781-5

**Published:** 2019-12-27

**Authors:** Konrad A. Szychowski, Jan Gmiński

**Affiliations:** Department of Clinical Biochemistry and Laboratory Diagnostics, Institute of Medical Sciences, University of Opole, Oleska 48, 45-052 Opole, Poland

**Keywords:** Biochemistry, Molecular biology, Medical research, Molecular medicine, Pathogenesis

## Abstract

Under physiological and pathological conditions, elastin is degraded to produce elastin-derived peptides (EDPs). EDPs are detected in the healthy human brain, and its concentration significantly increases after ischemic stroke. Both elastin and EDPs contains replications of the soluble VGVAPG hexapeptide, which has a broad range of biological activities. Effects of VGVAPG action are mainly mediated by elastin-binding protein (EBP), which is alternatively spliced, enzymatically inactive form of the *GLB1* gene. This study was conducted to elucidate the activation and role of the N-methyl-D-aspartate receptor (NMDAR) in elastin-derived VGVAPG peptide-dependent calcium homeostasis in mouse cortical astrocytes *in vitro*. Cells were exposed to 10 nM VGVAPG peptide and co-treated with MK-801, nifedipine, verapamil, or Src kinase inhibitor I. After cell stimulation, we measured Ca^2+^ level, ROS production, and mRNA expression. Moreover, the *Glb1* and NMDAR subunits (*GluN1, GluN2A*, and *GluN2B*) siRNA gene knockdown were applied. We found the VGVAPG peptide causes Ca^2+^ influx through the NMDA receptor in mouse astrocytes *in vitro*. Silencing of the *Glb1, GluN1, GluN2A*, and *GluN2B* gene prevented VGVAPG peptide-induced increase in Ca^2+^. Nifedipine does not completely reduce VGVAPG peptide-activated ROS production, whereas MK-801, verapamil, and Src inhibitor reduce VGVAPG peptide-activated Ca^2+^ influx and ROS production. These data suggest the role of Src kinase signal transduction from EBP to NMDAR. Moreover, the VGVAPG peptide affects the expression of NMDA receptor subunits.

## Introduction

Elastin is an insoluble, amorphous, hydrophobic protein with widespread presence in the walls of blood vessels as well as in the microvasculature of the normal human brain^1^. Under physiological and pathological conditions, elastin is degraded to generate elastin-derived peptides (EDPs)^2,3^. EDPs are detected in the healthy human brain, and its levels significantly increase after an ischemic stroke^4,5^. Elastin and EDPs contains replications of the soluble VGVAPG hexapeptide, which has a broad range of biological activities, such as increasing cell proliferation and migration, affecting chemotaxis, or causing protease release^6–8^. Effects of VGVAPG action are mainly mediated by elastin-binding protein (EBP), which is a alternatively spliced, enzymatically inactive form of the *GLB1* gene (β-galactosidase), known as S-Gal^9^.

In the central nervous system (CNS), astrocytes are responsible for the synthesis of extracellular matrix (ECM), supply of nutrients and oxygen to neurons, removal of dead neurons, and playing a role in neurotransmission and synaptic connections^10,11^. To date, it is known that astrocytes do not have action potentials. However, they display spontaneous and pharmacologically evoked increases in intracellular Ca^2+^ concentration ([Ca^2+^]i), which is a sign of its excitability^12^. In astrocytes, an increase in the Ca^2+^ level can result in mitochondrial dysfunction, increased production of free radicals, and activation of degenerative process controlled by Ca^2+^-activated proteases and phospholipases^13^. Therefore, it has been suggested that intracellular Ca^2+^ influx after trauma, ischemia, or stroke leads to cell damage^13,14^.

The N-methyl-D-aspartate (NMDA) receptor (NMDAR) is the most important excitatory receptor, permeable to Ca^2+^, Na^+^, and K^+^. However, its permeability to ions is strongly dependent on the composition of the subunit^15^. NMDAR are heterotetramers composed of two GluN1 subunits and two GluN2 A-D or GluN3 A-B subunits^16^. The di-heteromeric GluN1/GluN2B and GluN1/GluN2A receptors are an important fraction of juvenile and adult NMDARs. Moreover, in the adult CNS, particularly in structures such as the hippocampus and cortex, GluN2A and GluN2B are the predominant subunits^17,18^. It is well known that the NMDAR is involved in excitotoxicity-induced cell death in human embryonic stem cell-derived neurons, neuroblasts, and neuroblastoma cells^19–21^. NMDAR-mediated excitotoxicity is Ca^2+^-dependent and is typical of the nervous system^22^.

Studies to date have described that κ-elastin increases Ca^2+^ influx in human monocytes and fibroblasts, as well as in smooth muscle cells from pig aorta^23^. Similar, tropoelastin, EDPs, and the VGVAPG peptide increase the Ca^2+^ level in human umbilical venous endothelial cells (HUVEC)^24,25^. Moreover, in different glioma cell lines (C6, CB74, CB109, and CB191) κ-elastin or the (VGVAPG)_3_ peptide provoked a pronounced and dose-dependent increase in [Ca^2+^]i^26^.

It is well known that different Ca^2+^ signaling pathways interact with other cellular signaling systems such as reactive oxygen species (ROS)^27^. However, data on the ROS production in relation to EDPs are limited. To date, EDPs decrease ROS production in human neutrophils^28^. On the other hand, it has been described that EDPs induce ROS production in murine monocytes and human fibroblasts^29–31^. However, such data suggest that the effects of EDPs are cell and/or tissue dependent. In a previous study, we found that the VGVAPG peptide increases ROS production in an EBP-dependent manner both in mouse astrocytes *in vitro*^32^ and a human neuroblastoma (SH-SY5Y) cell line^33^. It is well known that calcium influx the NMDAR stimulates ROS production^34^. Moreover, signaling pathways transduced through the elastin receptor can use G protein and, thus, calcium channels are opened^35^. EBP-mediated cell proliferation has been described to involve the activation of Gi proteins with the opening of calcium L-type channels and resultant increase in Ca^2+^ levels in porcine smooth muscle cells^36^.

The present study was undertaken to elucidate the activation and role of the NMDAR in elastin-derived VGVAPG peptide-dependent calcium homeostasis in mouse cortical astrocytes *in vitro*.

## Results

### Ca^2+^ determination

**Figure 1:**
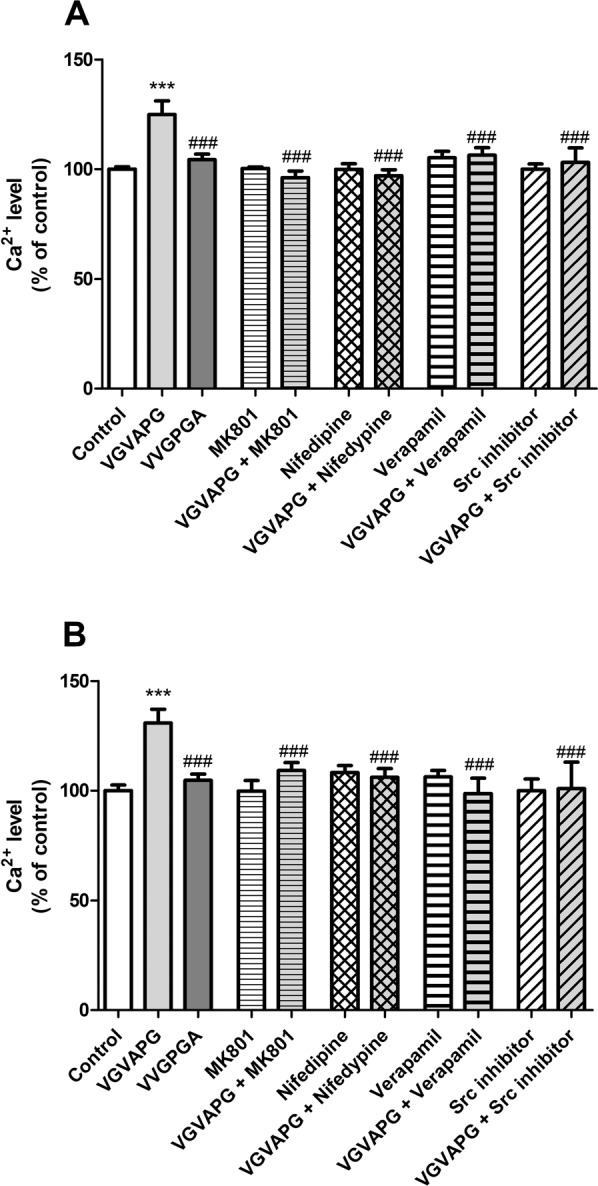
The effect of 10 nM VGVAPG peptide, 10 nM VVGPGA peptide, and co-treatment with 1 µM MK-801, 10 µM nifedipine, 10 µM verapamil, or 1 µM Src kinase inhibitor I on Ca^2+^ level in primary mouse astrocytes; (**A**) after 15 min and (**B**) after 30 min of exposure. Each point represents the mean ± SD of three independent experiments, each of which comprised six replicates per treatment group. *P < 0.05, **P < 0.01, and ***P < 0.001, versus the control cells. ^###^*P* < 0.001, versus the VGVAPG peptide-treated group.

After 15-min exposure of mouse astrocytes to 10 nM VGVAPG peptide, the level of cellular Ca^2+^ increased by 24.92% compared to control. VVGPGA does not cause influx of Ca^2+^ in to cells. MK-801, nifedipine, verapamil, and Src kinase inhibitor I prevented Ca^2+^ influx induced by the VGVAPG peptide (Fig. 1A).

After 30-min exposure of cells to VGVAPG peptide, the Ca^2+^ influx remained at a similar level as after 15-min exposure. The VGVAPG peptide increased of cellular Ca^2+^ level by 30.33% compared to control. VVGPGA does not cause influx of Ca^2+^ in to cells. MK-801, nifedipine, verapamil, and Src kinase inhibitor I prevented Ca^2+^ influx induced by VGVAPG peptide (Fig. 1).

### ROS measurement

**Figure 2:**
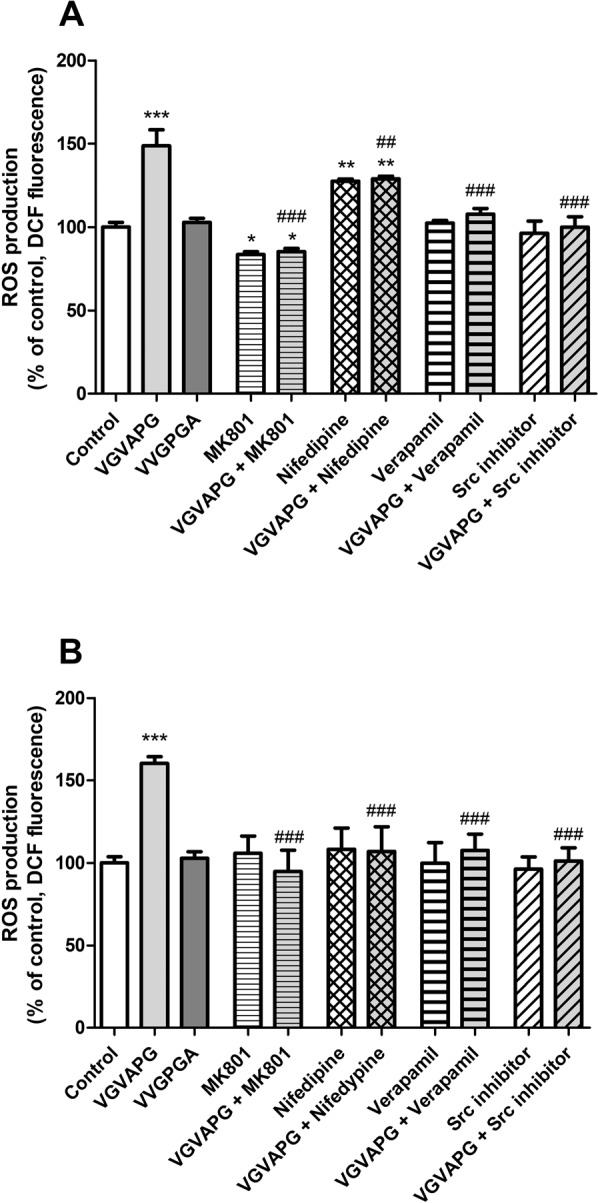
Effect of 10 nM VGVAPG peptide, 10 nM VVGPGA peptide, and co-treatment with 1 µM MK-801, 10 µM nifedipine, 10 µM verapamil, or 1 µM Src kinase inhibitor I on ROS production in primary mouse astrocytes; (**A**) after 3-h exposure and (**B**) after 6-h exposure. Each point represents the mean ± SD of three independent experiments, each of which comprised six replicates per treatment group. *P < 0.05, **P < 0.01, and ***P < 0.001, versus the control cells. ^##^*P* < 0.01 and ^###^*P* < 0.001, versus the VGVAPG peptide-treated group.

After 3-h exposure of mouse astrocytes to 10 nM VGVAPG peptide, we observed an increase in ROS production by 48.70% compared to the control. VVGPGA does not stimulate ROS production in astrocytes. MK-801 alone reduced ROS production by 16.36%, compared to control. Interestingly, nifedipine alone increased ROS production by 27.53%, compared to control. MK-801, verapamil, and Src kinase inhibitor I reduced ROS production stimulated by VGVAPG peptide to vehicle control levels. Nifedipine slightly but significantly reduced ROS production stimulated by the VGVAPG peptide by 19.65% as compared to the VGVAPG peptide-stimulated group (Fig. 2A).

After 6 h of astrocyte exposure to VGVAPG peptide, we observed an increase in ROS production by 60.28% compared to control. VVGPGA does not stimulate ROS production in astrocytes. MK-801, verapamil, nifedipine, and Src kinase inhibitor I reduce ROS production stimulated by the VGVAPG peptide (Fig. 2B).

### Ca^2+^ level and ROS production in astrocytes after gene silencing procedure

#### Ca^2+^ level

**Figure 3:**
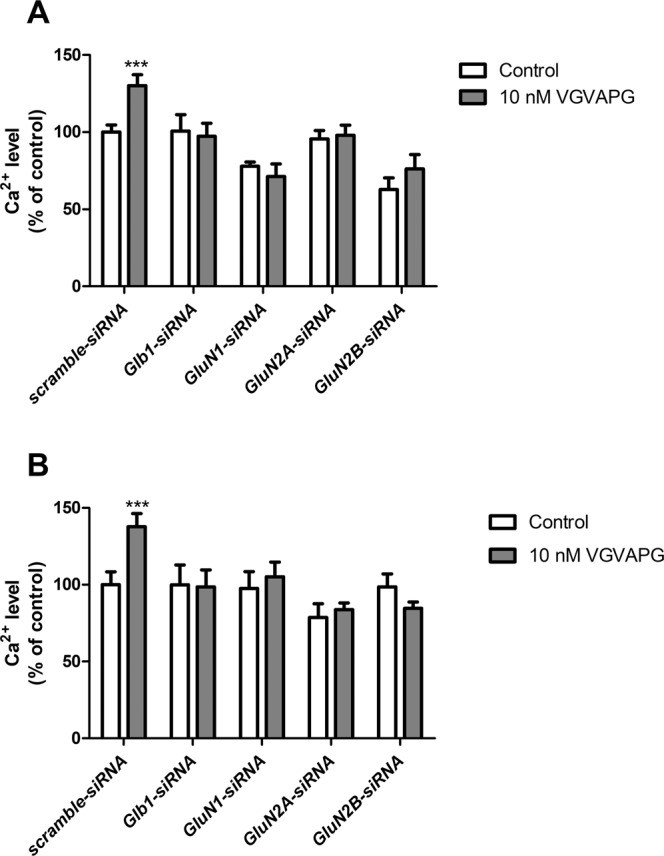
Effect of *GluN1*, *GluN2A*, and *GluN2B* siRNA on Ca^2+^ level after stimulation of primary mouse astrocytes with 10 nM VGVAPG peptide in primary mouse astrocytes; (**A**) after 15 min and (**B**) after 30 min of exposure. Each point represents the mean ± SD of three independent experiments, each of which comprised six replicates per treatment group. **P < 0.01 versus the control cells.

In cells transfected with scrambled siRNA, 15-min exposure to 10 nM VGVAPG peptide increased Ca^2+^ influx by 30.16% compared to control. The *Glb1* gene-silencing procedure protects cells by an increase in Ca^2+^ influx. Likewise, silencing of NMDAR subunits (*GluN1*, *GluN2A*, and *GluN2B*) protects cells by an increase in Ca^2+^ influx (Fig. 3A).

After 30-min exposure to VGVAPG peptide, results similar to those previously described were obtained. The VGVAPG peptide increased Ca^2+^ influx (increase by 37.69%) compared to control. Silencing of NMDAR subunits (*GluN1*, *GluN2A*, and *GluN2B*) protects cells by increasing Ca^2+^ influx (Fig. 3B).

#### ROS production

**Figure 4:**
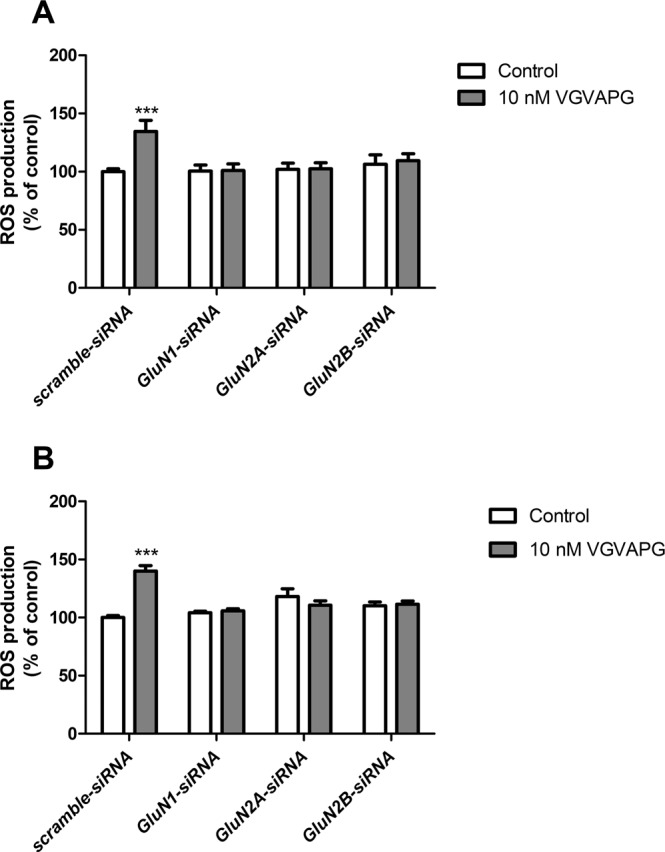
Effect of *GluN1*, *GluN2A*, and *GluN2B* siRNA on ROS production after stimulation of primary mouse astrocytes with 10 nM VGVAPG peptide; (**A**) after 3-h exposure and (**B**) after 6-h exposure. Each point represents the mean ± SD of three independent experiments, each of which comprised six replicates per treatment group. *P < 0.05, **P < 0.01, and ***P < 0.001, versus the control cells.

In cells transfected with scrambled siRNA, 3-h exposure to 10 nM VGVAPG peptide increased ROS production by 34.65% compared to the control. The *GluN1*, *GluN2A*, and *GluN2B* gene-silencing procedure protects cells by increasing ROS production (Fig. 4A).

After 6-h exposure to VGVAPG peptide, we obtained results similar to those that were previously described. VGVAPG peptide increases ROS production by 40.16% compared to the control. The *GluN1*, *GluN2A*, and *GluN2B* gene-silencing procedure protects cells by increased ROS production in astrocytes (Fig. 4B).

### mRNA expression of *GluN1*, *GluN2A*, and *GluN2B* NMDAR subunits

**Figure 5:**
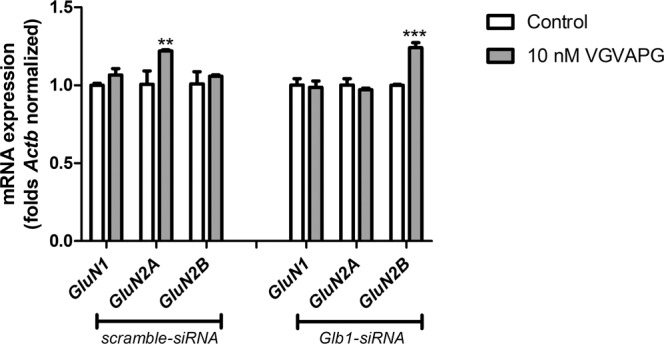
Effect of *Glb1* siRNA transfection on *GluN1*, *GluN2A*, and *GluN2B* mRNA expression after stimulation of primary mouse astrocytes with 10 nM VGVAPG peptide *in vitro*; after 6 h of exposure. Each point represents the mean ± SD of three independent experiments, each of which comprised six replicates per treatment group. **P < 0.01 and ***P < 0.001, versus the control cells.

In cells transfected with scrambled siRNA, 6-h exposure to 10 nM VGVAPG increased mRNA expression of the *GluN2A* NMDAR subunit by 21.66%. Silencing of the *Glb1* gene protects cells through the VGVAPG peptide, and we did not observe changes in mRNA expression of the *GluN1* and *GluN2A* genes. However, silencing of the *Glb1* gene causes 10 nM VGVAPG to induce an increase in mRNA expression of the *GluN2B* gene (increase by 24.16% compared to control; Fig. 5).

## Discussion

Our experiments are the first to show that the VGVAPG peptide increases Ca^2+^ influx in to mouse primary astrocytes *in vitro*. The experiments showed that MK-801 (a noncompetitive antagonist of the NMDAR) and Src kinase inhibitor I completely prevent Ca^2+^ influx after VGVAPG stimulation. Calcium-channel antagonists are a heterogeneous group of compounds. Nifedipine acts primarily on the smooth muscle cells of blood vessels, and has little effect on the heart. On the other hand, verapamil is cardioselective, and mainly affects the heart by blocking voltage-gated calcium channels^37^. Therefore, we chose nifedipine and verapamil for further research. Moreover, our study shows that nifedipine and verapamil prevent Ca^2+^ influx stimulated by the VGVAPG peptide in mouse astrocytes *in vitro*, although not as strongly as MK-801 or Src kinase inhibitor. Moreover, after silencing of both the *Glb1* gene and NMDAR subunits (*GluN1*, *GluN2A*, and *GluN2B* genes), the VGVAPG peptide does not activate Ca^2+^ influx. The data we obtained suggest that the activation of EBP results in opening of the NMDAR and other types of calcium channels are less often blocked by nifedipine and verapamil. To date, it is well documented that EDPs and/or the VGVAPG peptide initiate Ca^2+^ influx in different cell types such as HUVEC, SMC, fibroblasts, and gliomas^23–26,38^. Moreover, numerous reports have shown that the EDP-activated influx of Ca^2+^ into cells requires EBP activation^23–25,38^. Some authors have suggested that G proteins and c-Src as well as ERK1/2 or MEK1/2 kinases are involved in signal transduction, although the molecular mechanism mediating the opening of Ca^2+^ channels remains unclear^36,38,39^.

The next step of our study was to determine ROS production as a consequence of the influx of Ca^2+^ ions into cells. We found that stimulation of primary astrocytes by the VGVAPG peptide increased ROS production in both of the time intervals studied. MK-801, verapamil, and Src inhibitor prevent ROS production that was stimulated by the VGVAPG peptide. Similarly, after silencing of NMDAR subunits (*GluN1*, *GluN2A*, and *GluN2B* genes), the VGVAPG peptide did not cause ROS production. Interestingly, nifedipine does not completely prevent the ROS production stimulated by the VGVAPG peptide. Our results suggest that the binding of the VGVAPG peptide to EBP leads to increased ROS production, which mainly could be NMADR dependent. To date, reports concerning ROS production after EDP and/or VGVAPG stimulation are inconsistent. Our previous studies have shown that the VGVAPG peptide increases ROS production in an EBP-dependent manner in mouse primary astrocytes *in vitro*^32^. Similarly, an increase in ROS production after EDP stimulation has been reported in monocytes, human fibroblasts, and a neuroblastoma (SH-SY5Y) cell line^29,30,33^. Moreover, EDPs have been described to enhance the activities of antioxidant enzymes, such as superoxide dismutase (SOD), catalase (CAT), or glutathione peroxidase (GPx), and increase lipid peroxidation in human fibroblasts^40^. Furthermore, the VGVAPG peptide increases the expression and activity of GPx in SH-SY5Y cells^33^. On the other hand, the VGVAPG peptide reduced ROS production in neutrophils in controls and patients with stable chronic obstructive pulmonary disease^28^.

Phosphorylation regulates many cellular processes, including protein activity, localization, mobility, and protein–protein interactions. Direct phosphorylation of ionotropic glutamate receptors is a key mechanism that regulates channel function and receptor localization at synapses^41,42^. The proto-oncogene tyrosine-protein kinase Src is an important modulator of NMDARs. It has been described that the NR2A, NR2B, and NR2D subunits can be tyrosine phosphorylated^41^. Moreover, phosphorylation of the abovementioned subunits results in increased NMDAR activity^43^. Our data are the first to show that, in astrocytes *in vitro*, the Src kinase inhibitor I prevents Ca^2+^ influx and ROS production stimulated by the VGVAPG peptide. These data suggest a role of Src kinase in signal transduction from EBP to NMDAR and, probably, in other calcium channels.

It is well known that, in cells of the nervous system, the NMDAR is one of the most important ion channels^19–21^. NMDAR are responsible for Ca^2+^ influx and cellular ROS production that can result in excitotoxic cell death. However, our previous studies have shown that VGVAPG is nontoxic, and does not activate the apoptotic process in mouse astrocytes *in vitro*^44^. Therefore, we decided to determine the involvement of NMDAR subunits in the mechanism of action of VGVAPG in astrocytes *in vitro*. Our experiment with silencing of the *GluN1*, *GluN2A*, and *GluN2B* genes confirmed the experiments with the NMDAR antagonist MK-801. Moreover, we determined that the VGVAPG peptide affects the mRNA expression of *GluN2A* NMDAR subunits. In cells transfected with scramble siRNA, 10 nM VGVAPG peptide increased the mRNA expression of the *GluN2A* NMDAR subunit. On the other hand, after the silencing of the *Glb1* gene, we did not observe changes in the mRNA expression of *GluN2A* genes. However, silencing of the *Glb1* gene ensured the 10 nM VGVAPG could increase the mRNA expression of the *GluN2B* gene. The proportion of the GluN2B and GluN2A subunits changes with age and indicates the maturation of the nervous system^20,45^. In fetal life, strong expression of the GluN2B subunit is observed whereas, after birth, this expression decreases in favor of the GluN2A subunit^46,47^. However, a heterotetramer containing GluN2B subunits is mainly responsible for excitotoxicity and the intensity of neuronal apoptosis, whereas the opposite effect is caused by the presence of GluN2A subunits promoting cell survival^48,49^. Our data suggest that the VGVAPG peptide changes the NMDAR expression profile to one similar to the adult profile. Increased expression of *GluN2A* can promote cell survival and supports our hypothesis postulated in our other papers that the VGVAPG peptide promotes regeneration and the healing process in astrocytes^32,44,50^. However, after *Glb1* silencing, the NMDAR expression profile changed to similar to an embryonic one, and could promote cell apoptosis. Furthermore, these data suggest the involvement of other EDP receptors.

## Conclusion

Our study shows, for the first time, that the VGVAPG peptide causes Ca^2+^ influx through NMDAR in mouse astrocytes *in vitro*. Silencing of the *Glb1, GluN1, GluN2A*, and *GluN2B* genes prevented a VGVAPG peptide-induced increase in the Ca^2+^ level. Nifedipine does not completely reduce VGVAPG peptide-activated ROS production, whereas MK-801, verapamil, and Src kinase inhibitor I reduce VGVAPG peptide-activated Ca^2+^ influx and ROS production. There appears to be a potential role of Src kinase in signal transduction from EBP to NMDAR. Moreover, the VGVAPG peptide induced an increase in the expression of *GluN2A* NMDAR subunits, which promote cell survival in adult life. However, due to the lack of sufficient data explaining the molecular mechanism of action of the VGVAPG peptide in the nervous system, more studies on this topic are necessary.

## Materials and Methods

### Reagents

We purchased Dulbecco’s Modified Eagle’s Medium/Hams F-12 (DMEM/F12) without phenol red, along with trypsin, rosiglitazone, pioglitazone, penicillin, streptomycin, amphotericin B, nifedipine, verapamil, MK-801, and dimethyl sulfoxide (DMSO) from Sigma–Aldrich (St. Louis, MO, USA). *Glb1* gene siRNA (sc-61342), *NMDAζ1 (GluN1)* (sc-36082), *NMDAε1 (GluN2A)* (sc-36084), and *NMDAε2 (GluN2B)* (sc-36086) gene siRNA were purchased from Santa Cruz Biotechnology (Santa Cruz, CA, USA). The INTERFERin siRNA transfection reagent was purchased from Polyplus-transfection (Illkirch, France). The VGVAPG and VVGPGA peptides were synthesized by LipoPharm.pl (Gdańsk, Poland). Charcoal/dextran-treated fetal bovine serum (FBS) was purchased from EURx (Gdańsk, Poland). The Src kinase inhibitor I (14592) and Fluo-3 AM (14960) were purchased from the Cayman Chemical Company (Michigan, USA). The High Capacity cDNA – Reverse Transcription Kit and the TaqMan probes corresponding to specific genes encoding *Actb* (Mm00607939_s1), *GluN1* (Mm00433800_m1), *GluN2A* (Mm00433802_m1), and *GluN2B* (Mm00433820_m1) were obtained from Life Technologies Applied Biosystems (Foster City, CA, USA). Stock solutions of the VGVAPG and VVGPGA peptides were prepared in DMSO, and then added to the DMEM/F12 medium. The final concentration of DMSO in the culture medium was always 0.1%.

### Astrocyte-enriched cell culture

All experiments were conducted on mouse astrocyte cell cultures, which were prepared from the fetuses of pregnant female Swiss mice according to a previously described method that generates an almost pure culture of astrocytes (>98% astrocytes)^44^(supplementary data). Cells were cultured in DMEM/F12 1:1 without phenol red that was supplemented with 10% FBS, 100 U/mL penicillin, 0.10 mg/mL streptomycin, and 250 ng/mL amphotericin B, maintained at 37 °C in a humidified atmosphere containing 5% CO_2_. The astrocytes were seeded at a density of 5 × 10^5^ per well in a 96-well plate, for colorimetric and fluorometric analysis, and 60 × 10^5^ per well in a 12-well plate for mRNA analysis. The culture medium was changed prior to the experiment with the VGVAPG peptide and a tool compound was selected for this study.

### siRNA gene-silencing procedure

*Glb1, GluN1, GluN2A*, and *GluN2B* siRNA were used to inhibit the expression of EBP and NMDAR subunits in mouse primary astrocytes. The experiment was conducted in accordance with a previously described procedure^51^. Briefly, appropriate siRNA was applied for 7 h at a final concentration of 50 nM in antibiotic-free medium containing the siRNA transfection reagent INTERFERin. After transfection, the astrocytes were cultured for 12 h prior to commencing the experiment. Vehicle controls included positive siRNA and scramble siRNA containing a scrambled sequence that did not lead to specific degradation of any known cellular mRNA. Knockdown of the *Glb1* gene was estimated at 39% of the control mRNA and 60.05% of the control protein as previously described^44,50^. Knockdown of the *GluN1, GluN2A*, or *GluN2B* genes were estimated at 22.02, 25.21 and 24.32%, respectively of the control mRNA.

### Ca^2+^ determination in astrocytes

To measure the calcium level, we used the Fluo-3/AM indicator, which exhibits increased fluorescence emission intensity on binding to Ca^2+^ and, therefore, is used to detect increases in the level of intracellular calcium^52^. Briefly, a final concentration of 4 μM Fluo-3/AM dye and 0.04% Pluronic F-127 in the loading medium (DMEM/F12 without FBS) was used for 60 min before compound treatment. Thereafter, the cells were washed three times with 200 μL PBS without Ca^2+^ supplemented with HEPES. After 15 and 30 min of incubation of the cells with 10 nM VGVAPG or VVGPGA peptide (5% CO_2_ at 37 °C), the fluorescence of Fluo-3 was measured with a microplate reader (FilterMax F5) at maximum excitation and emission spectra of 485 and 535 nm, respectively.

### ROS measurement

The fluorogenic dye H_2_DCFDA was used to detect intracellular ROS according to a previously described method^53^. Briefly, a final concentration of 5 μM H_2_DCFDA was used. The cells were incubated in H_2_DCFDA in a serum-free and phenol red-free DMEM/F12 medium for 45 min before compound treatment. After 3- and 6-h incubation of the cells with 10 nM VGVAPG or VVGPGA peptide (5% CO_2_ at 37 °C), the culture medium was replaced with fresh medium to remove the extracellular residual DCF and the studied compound to reduce the fluorescence background. Cells treated with 0.3% hydrogen peroxide (H_2_O_2_) were used as a positive control (results not shown). DCF fluorescence was measured with a microplate reader (FilterMax F5) at maximum excitation and with emission spectra of 485 and 535 nm, respectively.

### Real-time PCR analysis of mRNAs specific to genes encoding *GluN1, GluN2A*, and *GluN2B*

The experiment was conducted through a previously described procedure^54^. For the real-time PCR assay, astrocytes were seeded onto 12-well plates and initially cultured for 24 h. After 6-h exposure to 10 nM VGVAPG peptide, the samples were collected, and total RNA was extracted from the astrocytes using a Qiagen RNeasy Mini Kit according to the manufacturer’s instructions. Both the quality and quantity of the RNA were determined spectrophotometrically at 260 and 280 nm, respectively (ND/1000 UV/Vis; Thermo Fisher NanoDrop, USA). Two-step real-time reverse transcription (RT)-PCR was conducted with both the RT reaction and the quantitative PCR (qPCR) run using the CFX Real Time System (BioRad, USA). The RT reaction was carried out at a final volume of 20 μL with 180 ng RNA (as a cDNA template) using the cDNA reverse transcription kit in accordance with the manufacturer’s instructions. Products from the RT reaction were amplified using the FastStart Universal Probe Master (Rox) kit with TaqMan probes as primers for specific genes encoding *Actb, Glb1, GluN1, GluN2A*, and *GluN2B*. Amplification was carried out in a total volume of 20 μL containing 1× FastStart Universal Probe Master (Rox) and 1.5 μL of the RT product, which was used as the PCR template. Standard qPCR procedures were carried out as follows: 2 min at 50 °C and 10 min at 95 °C, followed by 45 cycles of 15 s at 95 °C and 1 min at 60 °C. The threshold value (Ct) for each sample was set during the exponential phase, and the ΔΔCt method was used for data analysis. *Actb* was used as the reference gene. Random-peptide-sequence VVGPGA was used as control and did not affect *GluN1, GluN2A*, and *GluN2B* mRNA expression (data not shown).

### Statistical analysis

Data are presented as means ± SD of three independent experiments. Each treatment was repeated six times (n = 6) and measured in triplicate. Data were analyzed using Multi-Mode Analysis STATISTICA 13.0 software and normalized to fluorescence in the vehicle-treated cells (% of control). One-way analysis of variance (ANOVA) followed by Tukey’s multiple comparison procedure was applied, with ***p < 0.001, **p < 0.01, and *p < 0.05 indicative of statistical significance versus the control.

### Ethical approval

Animal care followed official governmental guidelines, and all efforts were made to minimize the number of animals used and their suffering. All procedures were performed in accordance with the National Institutes of Health Guidelines for the Care. Moreover, all procedures were approved by a Bioethics Commission (no. 46/2014, First Local Ethical Committee on Animal Testing at the Jagiellonian University in Krakow), as compliant with the European Union law.
